# Enhanced toxicity to chemoradiation in a patient with Anti-Jo-1-antisynthetase syndrome

**DOI:** 10.1259/bjrcr.20210188

**Published:** 2022-02-24

**Authors:** Luca Valle, James Katz, Austin Duffy, Matthew Hueman, Hao-Wei Wang, Marybeth Hughes, Tristan Sissung, William Figg, Deborah Citrin

**Affiliations:** 1 Department of Radiation Oncology, University of California Los Angeles, Los Angeles, CA, USA; 2 National Institutes of Arthritis and Musculoskeletal and Skin Diseases, Bethesda, MD, USA; 3 Thoracic and Gastrointestinal Oncology Branch, Center for Cancer Research, National Cancer Institute, Bethesda, MD, USA; 4 Surgical Oncology Division, Murtha Cancer Center, Walter Reed National Military Medical Center, Bethesda, MD, USA; 5 Laboratory of Pathology, Center for Cancer Research, National Cancer Institute, Bethesda, MD, USA; 6 Division of Surgical Oncology, Department of Surgery, Eastern Virginia Medical School, Norfolk, VA, USA; 7 Genitourinary Malignancies Branch, Center for Cancer Research, National Cancer Institute, Bethesda, MD, USA; 8 Radiation Oncology Branch, Center for Cancer Research, National Cancer Institute, Bethesda, MD, USA

## Abstract

Appropriate counseling of patients with autoimmune connective tissue disorders (ACTDs) is often challenging for radiation oncologists, especially regarding anticipated side-effects of radiation treatment. These patients can have highly variable and unpredictable sequelae from radiation therapy, and the uncertainty builds when radiation is convoluted by the addition of concurrent chemotherapy. While many patients may experience a mild intensification of toxicity above what is expected, some patients experience much more severe toxicity. These patients become critical learning cases, enabling a better understanding of the delicate and complex ways in which radiation response is altered in the context of ACTDs while allowing other patients with similar ACTD profiles to benefit from past experience. Our report makes an important contribution to this space by describing a particularly severe case of toxicity that manifested in such a patient and the ensuing clinical decision-making. Comprehensive genotyping of classic pharmacokinetic and pharmacodynamic pathway genes (including mutations in DPD and CDA) did not reveal any signatures that might explain her enhanced toxicity and we demonstrate that severe toxicity can still manifest in the era of modern conformal radiation treatments for rectal cancer. We urge caution in the treatment of patients with rare ACTDs, but also emphasize that curative treatment should not be withheld in such patients. We conclude by advocating for the development and maintenance of a prospective multiinstitutional database of patients with ACTDs to help inform and improve future practice.

## Introduction

The impact of comorbid autoimmune connective tissue disorders (ACTDs) on radiation toxicity is uncertain. However, because some studies have demonstrated an increased risk of radiation toxicity in patients with ACTDs^1,2^ the presence of some ACTDs is considered a relative contradiction to radiation therapy.^3^ Anti-Jo-1-antisynthetase syndrome (AJ1AS) is an ACTD defined by the presence of serum antibodies to a specific aminoacyl-transfer RNA synthetase. It is an idiopathic inflammatory subtype of dermatomyositis with symptoms including fever, interstitial lung disease, Raynaud’s phenomenon, and polyarthritis.^4^

The association between dermatomyositis and malignancy is well-established.^5^ Case studies of AJ1AS have reported various cancers occurring within 6 months of antisynthetase syndrome diagnosis,^6^ fueling speculation that AJIAS might be paraneoplastic in etiology. Given the link between ACTDs and cancer as well as increasing survival of patients harboring ACTDs,^7^ the number of these patients who will require radiation is likely to increase.

This report details a case of severe chemoradiation toxicity in a patient with AJ1AS. Given the paucity of published reports describing an association between AJ1AS and treatment toxicity, we sought to describe the management and clinical decision-making surrounding her case, review the current knowledge regarding treatment toxicity with comorbid ACTDs, and suggest best practices when considering radiotherapy for patients with rare ACTDs.

## Clinical presentation

**Figure 1.**
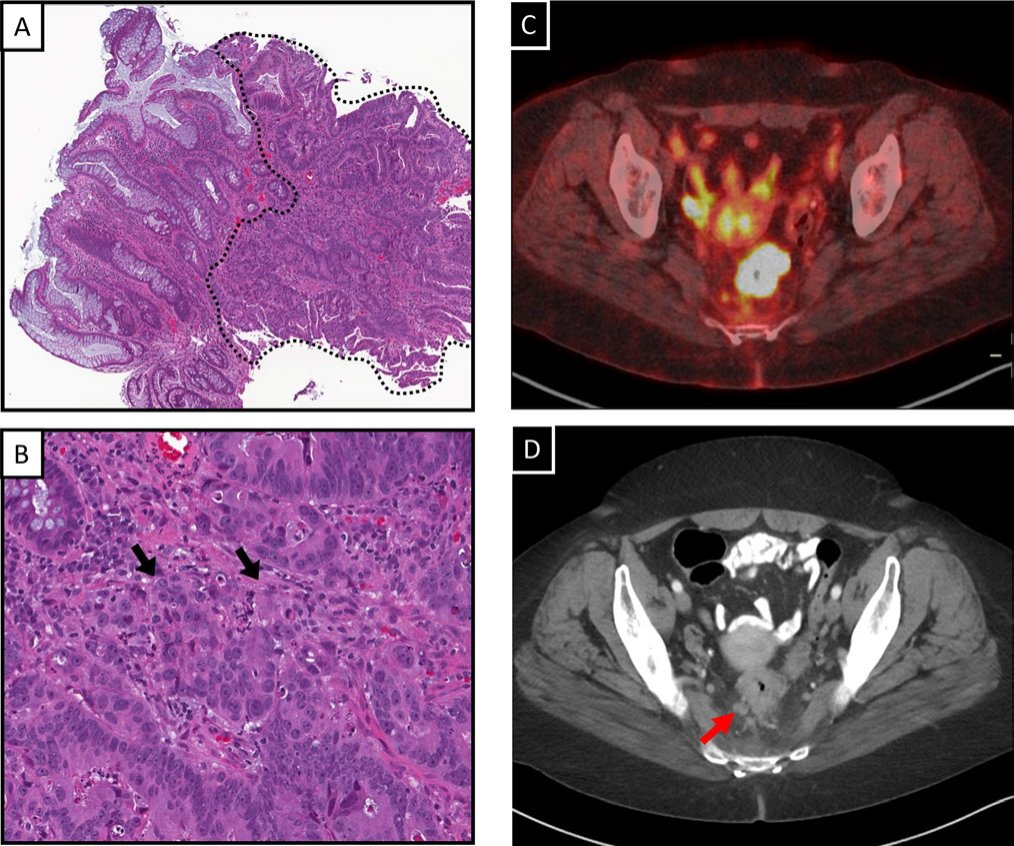
Pre-treatment biopsy and imaging. Low (**A**) and high (**B**) power H&E stains of the rectal mass. The adenocarcinoma is outlined by dotted black line (**A**), invasion into the lamina propria is indicated by arrows (**B**). Pre-treatment PET-CT (**C**)and CT Pelvis (**D**) demonstrate a circumferential thickening of the rectum and associated pathologic lymph node (red arrow).

A 44-year-old African American female presented with several weeks of blood-tinged stool and tenesmus. Colonoscopy revealed a circumferential 7 cm mass extending from the rectosigmoid to 6.7 cm from the anal verge. Biopsy confirmed moderately differentiated invasive adenocarcinoma (Figure 1A and B). Baseline imaging identified extension into the perirectal fat and three pathologic perirectal lymph nodes (Figure 1C and D), resulting in the diagnosis of a clinical stage T3N1bM0 rectal adenocarcinoma.

**Figure 2.**
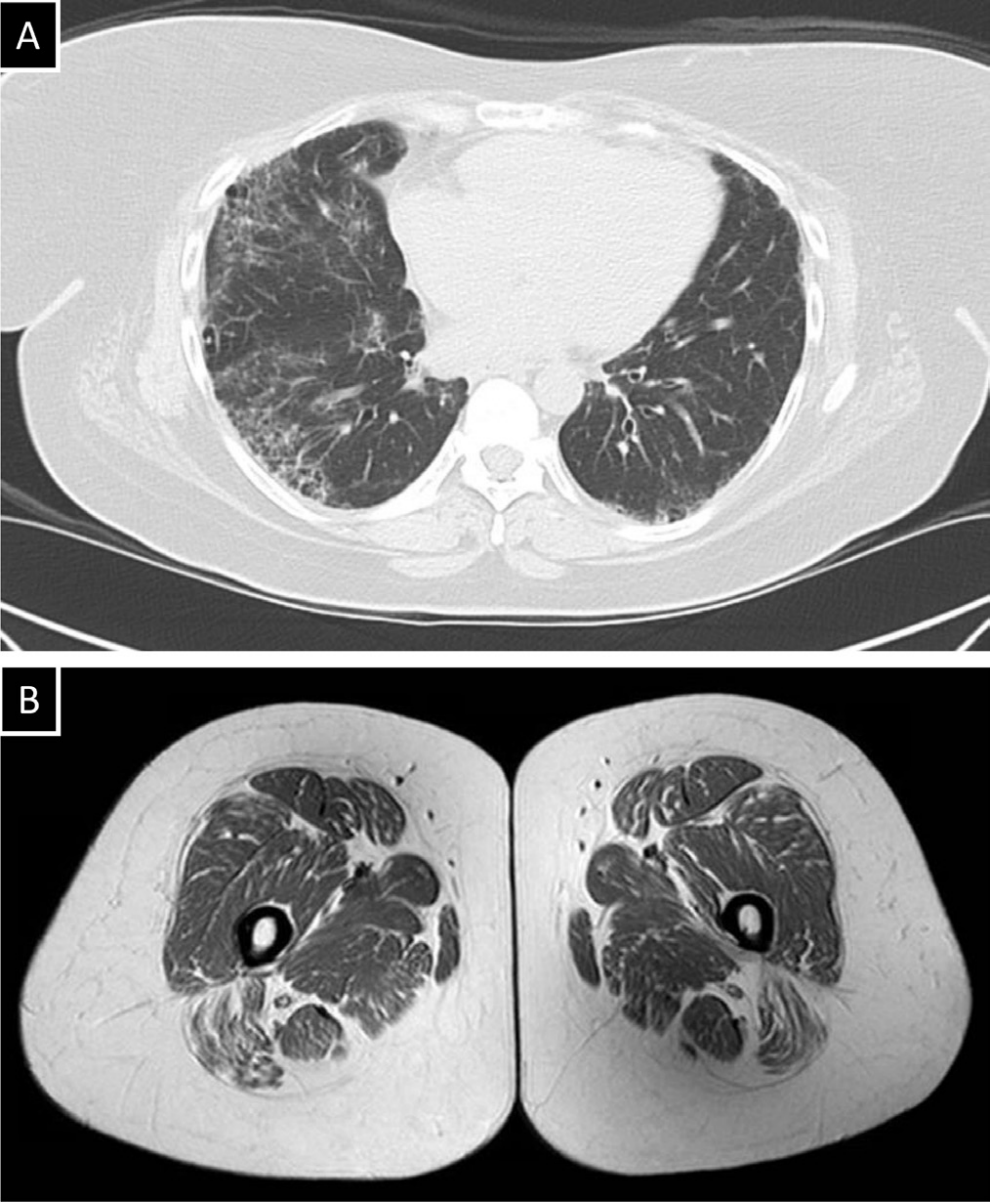
Anti-Jo-1 antisynthetase syndrome. (**A**) Chest CT demonstrating interstitial lung fibrosis and scattered pneumatocysts. (**B**) Lower extremity MRI revealing fatty infiltration of gluteal and quadriceps muscles.

Her past medical history was notable for AJ1AS with interstitial pulmonary fibrosis (Figure 2A), diagnosed 18 years prior and characterized by recurrent flares of extremity weakness and pain due to myositis with fatty infiltration (Figure 2B), later confirmed by biopsy.

The patient’s rectal cancer was treated with neoadjuvant capecitabine and radiation. intensity modulated radiation therapy (IMRT) was delivered to a dose of 45 Gy in 25 fractions to the regional lymph nodes and tumor with margin. A sequential boost of 5.4 Gy in 3 fractions was delivered to the presacral hollow and tumor with a 5 mm PTV margin to a total dose of 50.4 Gy. Immediately after initiating capecitabine at a dose of 1650 mg BID, the patient experienced severe nausea that was minimally responsive to antiemetics. At a dose of 36 Gy, a treatment break and hospitalization were required to manage intractable nausea and diarrhea. At that time, she was noted to have an elevated total and direct bilirubin, macrocytic anemia requiring transfusion, and afebrile neutropenia. After a 6-day treatment break, she resumed radiation and dose-reduced capecitabine at a dose of 1300 mg BID. Four days later, she permanently discontinued capecitabine due to intractable nausea. Her radiation course was completed to the prescribed dose.

Genetic testing using DNA obtained from whole blood was conducted via the Pharmacoscan^®^ array (Thermo, CA), which revealed that she was wild-type for cytidine deaminase (CDA*1/*1) and dihydropyrimidine dehydrogenase (DYPD*1/*1), genes important in the bioactivation and inactivation of capecitabine and 5-FU, respectively (Table 1, Supplementary Material 1). She was, however, a carrier for the 222Ala-429Glu and 222Val-429Glu diplotype in methylenetetrahydrofolate reductase, an enzyme that antagonizes capecitabine-induced thymidylate synthase inhibition.^8^

Supplementary Material 1.

**Table 1.** Genotype or diplotype of pharmacokinetic/pharmacodynamic pathway genes

rsID*		Genotype	Change for variant	Phenotype association	PMID
***CES1***				Normal capecitabine bioactivation	32458030
	N/A	All alleles are ref/ref	N/A		
***CES2***				Normal capecitabine bioactivation	18473752
	rs4783745	All alleles are ref/ref	Intronic	Unknown	
***CDA***				Normal capecitabine bioactivation	19107485
	N/A	CDA*1/*1	N/A		
***DPYD***				Normal 5-FU metabolism	29152729
	N/A	DPYD*1/*1	N/A		
***ABCC3***				Possible decreased transport	31186779
	rs4148416	C/T	G1013G	Poor response to chemotherapy	24996541
	rs2277624	C/T	H1314H	Unknown	
***ABCC4***				Possible decreased transport	28765596
	rs9516519	A/C	3’UTR	Poor methotrexate elimination	23222202
	rs4148551	T/C	3’UTR	Unknown	
	rs9556455	C/T		Unknown	
	rs2274406	G/G	R317R	Unknown	
	rs2274407	G/T	K304N	Aberrant mRNA splicing	
	rs7317112	T/C		Methotrexate toxicity	25348617
	rs868853	G/A	5’UTR	Unknown	
***ABCC5***				Unclear	
	rs1000002	C/T	3’UTR	Unknown	
	rs3749442	C/T	L1208L	Unknown	
	rs3792581	G/T		Unknown	
	rs1053386	C/T	S400S	Unknown	
	rs2293001	G/A		Lower mercaptopurine dose	26332308
	rs4148572	C/G		Unknown	
***ABCG2***				Normal transport	24338217
	rs2622628	T/G		Unknown	
	rs17731799	C/A		Unknown	
***SLC22A7 (OAT2)***				Unclear	28347776
	rs2270860	C/T	S425S	Skin toxicity in homozygous variants	28347776
***SLC29A1***					
	rs9394992	C/T		Neutropenia risk in heterozygous and homozygous variants	25162786
***MTHFR***				Altered FU disposition	23407049
	rs1801133	677 C/T	222 Ala/Val	Decreased MTHFR activity, thermolabile variant	
	rs1801131	A/A	429 Glu/Glu	Decreased MTHFR activity, increased gene methylation	
	665*C* > T-1406*A* > C diplotype	CA-TA	222Ala-429Glu 222Val-429Glu	High risk of diarrhea and mucositis with capecitabine and radiotherapy	23407049
***TYMS***				Possible altered FU disposition	23407049
	rs151264360	Del TTAAAG	3’-UTR	mRNA instability and reduced TYMS expression	29085228

FU, fluorouracil; MTHFR, methylenetetrahydrofolate reductase.

* Variant alleles are listed for each gene, and all data are taken from the supplemental “Pharmacoscan results” table.

**Figure 3.**
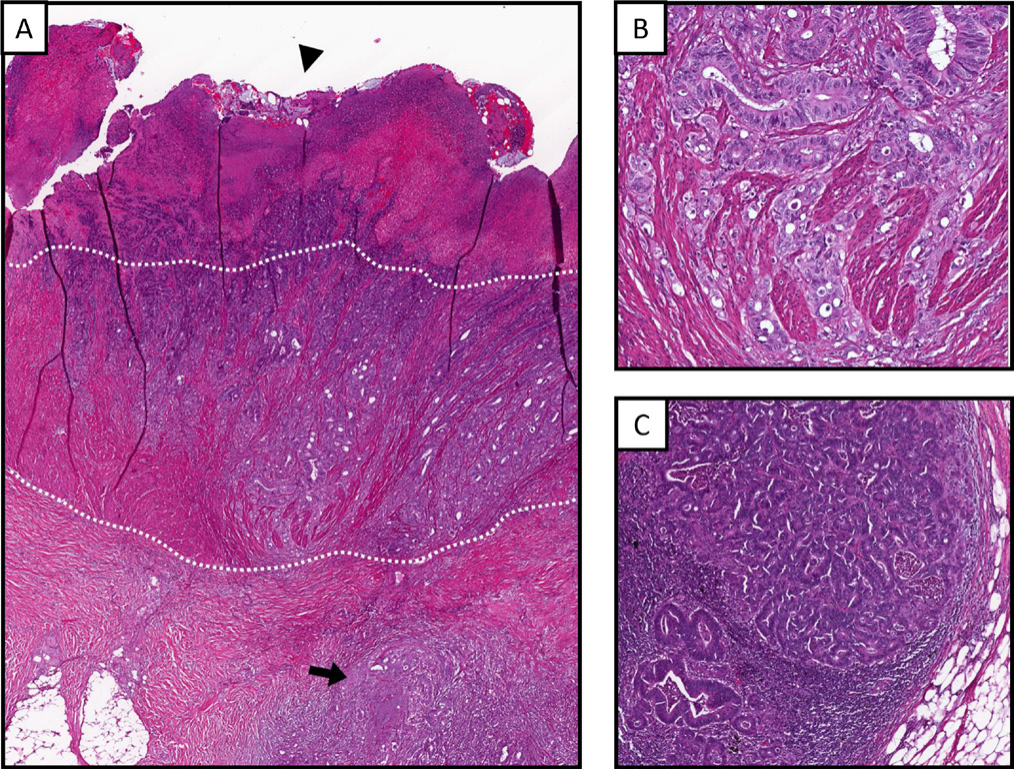
Surgical pathology. (**A**) H&E stain of the low anterior resection specimen shows a moderately to poorly differentiated adenocarcinoma with an ulcerative surface (arrow head), invading through the muscularis propria (outlined by dotted white lines) to involve the perirectal adipose tissue (arrow). (**B**) Higher magnification highlights the carcinoma invading through the muscularis propria. (**C**) Representative section of metastatic adenocarcinoma involving the mesenteric lymph node.

After completing chemoradiation, her nausea and vomiting continued. Pre-operative restaging CT revealed minimal tumor response 7 weeks after completing neoadjuvant therapy. She subsequently underwent low anterior resection of her tumor with a diverting ileostomy. During the operation, she was noted to have significant pelvic inflammation (Supplementary Material 1). The final surgical pathology revealed a high-grade, poorly differentiated pT3N2b adenocarcinoma with 11/16 lymph nodes involved (Figure 3).

**Figure 4.**
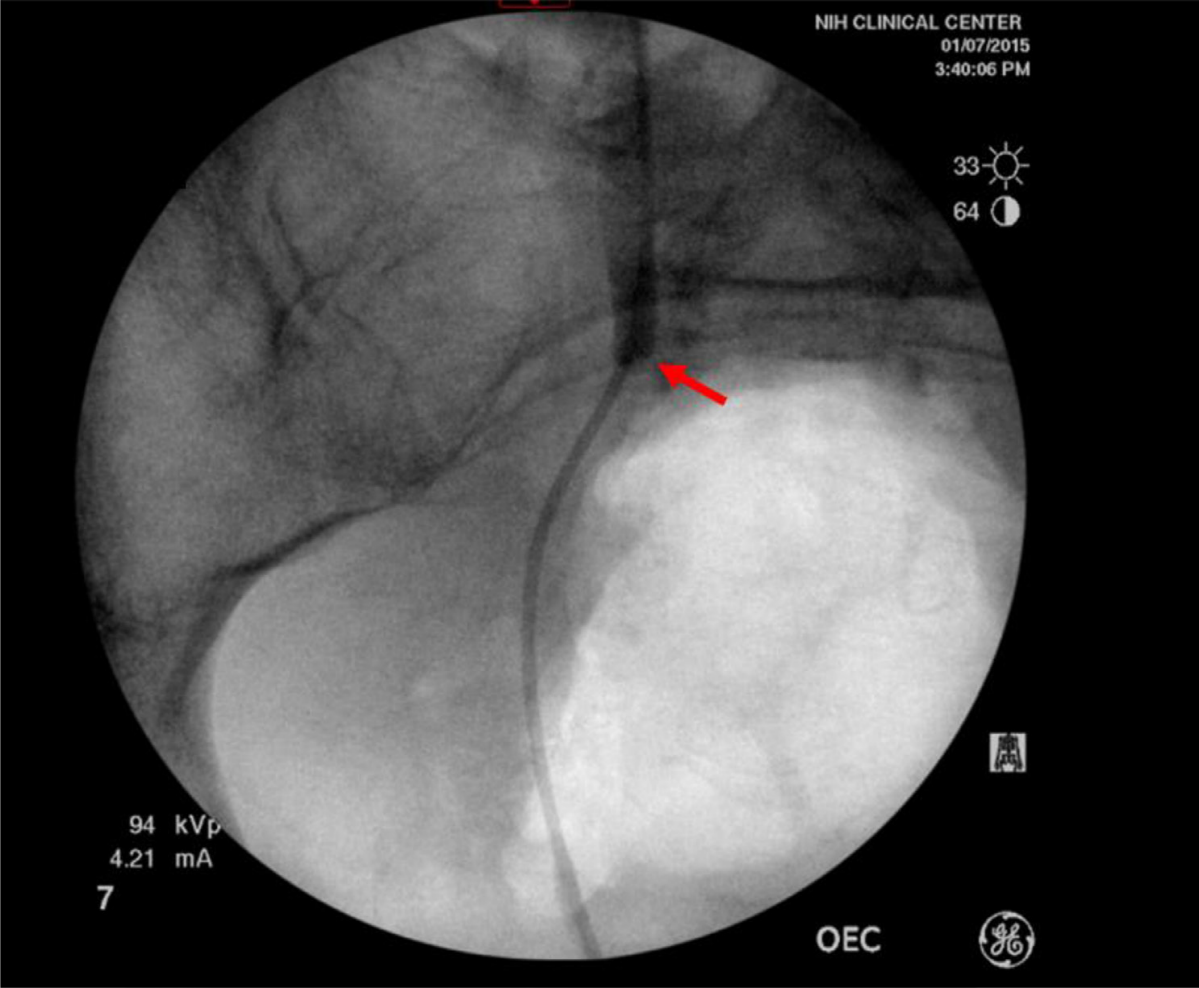
Post-treatment ureteral stricture. Ureteral obstruction isolated to the upper border of the radiation field (red arrow) viewed from a retrograde pyelogram.

Post-operatively, she developed a pre-sacral abscess requiring drainage. One month after surgery, she was noted to have unilateral hydronephrosis due to a ureteral stricture at the superior border of the radiation field (Figure 4). Her post-treatment course was further complicated by multiple hospitalizations for right flank pain, gross hematuria, intermittent bleeding per ostomy, and a small bowel obstruction amenable to medical management, all despite curtailing her adjuvant chemotherapy to two cycles of FOLFOX. Endoscopies did not reveal a source of bleeding. Adjuvant chemotherapy was abandoned due to delays from managing toxicity. The patient ultimately passed away from disease progression. metastatic progression of disease. Consent for publication of this case could not be obtained from the patient or next of kin despite exhaustive attempts.

## Evidence-based discussion

The advent of modern radiation delivery techniques has generated optimism that irradiation of patients with ACTDs may be less toxic due to increased sparing of normal tissue.^9^ Our report highlights the remarkable toxicity that can still manifest in ACTD patients treated with modern conformal radiation approaches. Enhanced radiation toxicity in this patient may involve an interaction with ionizing radiation and the immunological underpinings of AJ1AS. The pathophysiology of dermatomyositis is thought to include polyclonal B-cell activation, increased immunogenicity of autoantigens secondary to a primary site of inflammation, autophagy with activation of the NF-kB pathway, and a globally increased cytokine response.^10^ Many such mechanims are involved in the response to ionizing radiation as well,^11^ and the immune enhancement from radiation therapy might also result in abscopal autoimmune reactions at remote sites. Further study of the effects of exposure to ionizing radation beyond double-stranded DNA breaks, specifically as pertains to the population of patients with connective tissue disease, may be worthwhile.

In studies examining the correlation of ACTDs with severe radiation toxicity, risk depended largely on the type of ACTD, with scleroderma highly associated with radiation toxicity, rheumatoid arthritis not associated with increased toxicity, and other ACTDs falling somewhere in between.^12^ It has been hypothesized that ACTDs involving vascular inflammation may predispose for severe radiation toxicity due to additive radiation effects on vascular remodeling, intimal hyperplasia, and basement membrane exposure.^13^ As the pathophysiology of AJ1AS is not fully elucidated, it is unclear where it falls on this risk spectrum.

It is possible that severity of the ACTD at the time of treatment may predict toxicity.^14^ However, our patient experienced substantial toxicity even when undergoing radiation with what appeared to be clinically stable AJ1AS. Thus, outward clinical manifestations of rare ACTDs may not be a suitable barometer for anticipating toxicity risk, especially for ACTDs in which disease stability is difficult to monitor.

The location of radiation treatment may also play an important role in the likelihood of radiation toxicity in patients with ACTDs. Toxicity tends to be more common when treating the pelvis compared to other disease sites, and one series described over one-third of all patients treated with RT to the pelvis experiencing severe acute and late toxicity in the setting of ACTDs^2^

Regarding capecitabine-related toxicity, a correlation between fluoropyrimidine intolerance and AJ1AS has not been reported. However, the intractable vomiting and metabolic derangements we describe are likely related to capecitabine, given their temporal relation to drug dosing and the known emetic profile of thymidylate synthase inhibitors and their prodrugs. It remains unclear whether the radiation sequelae in this patient were related to an inherent sensitivity to radiation alone or a combination of therapeutic agents. The use of chemotherapy concurrent with radiotherapy has been associated with an increase in the rate of both acute and late toxicity in patients with ACTDs,^2^ and thus it is possible that impaired clearance of capecitabine could lead to both intrinsic drug toxicity as well as enhanced radiation toxicity through greater normal tissue exposure and ensuing radiosensitization.^15^

Genetic factors resulting in increased capecitabine toxicity may also affect outcome. Although the patient carried wild-type *DPD* and *CDA* genotypes, she also carried the 222Ala-429Glu and 222Val-429Glu diplotype (Table 1) associated with a higher likelihood of diarrhea and mucositis in patients receiving concurrent 5-FU and radiotherapy.^8^ The mechanism underlying this association is unclear, but may involve individual variation in MTHFR-mediated depletion of 5,10 methylenetetrahydrofolate, which is required for optimal thymidylate synthase inhibition. She also carried the TTAAAG deletion in TYMS, which potentially results in reduced TYMS expression.^16^

Cases of severe toxicity such as this provoke hesitation for employing radiation in the setting of ACTDs. A careful consideration of the risks and benefits of treatment must be undertaken on a case-by-case basis. Multidisciplinary management, including collaboration with rheumatology, is critical to optimize management of comorbid disease and medications that may impact toxicity. Radiation dose reduction must be approached cautiously in patients with ACTDs, as our case demonstrates that enhanced normal tissue sensitivity does not necessarily correlate to enhanced tumor sensitivity. Caution should also be employed when using hypofractionation or immune modulating agents in combination with radiation in patients with ACTDs, given the theoretical increased risk of late effects. Finally, we advocate for efforts to maintain a prospective multi-institutional database of patients with ACTDs undergoing chemotherapy and/or radiation to help inform future practice. It is our hope that these recommendations will aid providers and their patients in more accurately anticipating toxicity, better understanding the nature of toxicity, and more closely matching patient expectations with likely outcomes.

## Learning points

- Amidst optimism^9^ surrounding the potential to limit toxicity in ACTD patients with modern radiation techniques, patients with AJ1AS may have enhanced sensitivity to the toxicities of capecitabine and radiation, even in the modern treatment era.
- Life-saving cancer treatments, including radiation and chemotherapy, should not be withheld from patients with poorly understood ACTDs
- We advocate for efforts to maintain a prospective multi-institutional database of patients with ACTDs undergoing chemotherapy and/or radiation to help inform future practice.
