# Supplementary material for: Enhanced toxicity to chemoradiation in a patient with Anti-Jo-1-antisynthetase syndrome
Source: BJR Case Rep. 2022 Feb 24;8(3):20210188. doi: 10.1259/bjrcr.20210188 (PMC9461731; doi:10.1259/bjrcr.20210188)
Supplement: Supplementary Material 1. [file bjrcr.20210188.suppl-01.docx]

**Supplemental Table 1: Pharmacoscan Results**

| **rsID** | | **Nucleotide change** | **Genotype** |
| --- | --- | --- | --- |
| *CES1* | |  |  |
|  | rs3785161 | CES1P1:n.-816A>C | A/A |
|  | rs71647871 | CES1:c.431G>A | G/G |
|  | rs2307240 | CES1:c.227G>A:p.Ser76Asn | G/G |
|  | rs3815583 | CES1:c.-75T>G | T/T |
| *CES2* | |  |  |
|  | rs11075646 | CES2_c.-171C>G(5'UTR) | C/C |
|  | rs11568314 | CES2_c.268+947A>T | A/A |
|  | rs4783745 | CES2_c.269-965A>G | A/G |
|  | rs11568311 | CES2_c.269-683G>A | G/G |
|  | rs72547531 | CES2_c.292C>T(R98W) | C/C |
|  | rs72547532 | CES2_c.616G>A(V206M) | G/G |
|  | rs72547533 | CES2_c.1330-2A>G(SpliceDefect) | A/A |
| *CDA* | |  |  |
|  | rs532545 | CDA_c.-451C>T(5'UTR) | C/C |
|  | rs602950 | CDA_c.-92A>G(5'UTR) | A/A |
|  | rs602946 | CDA_c.-88G>A(5'UTR) | G/G |
|  | rs3215400 | CDA_c.-33delC(5'UTR) | -/C |
|  | rs2072671 | CDA*2_c.79A>C(K27Q) | A/A |
|  | rs818202 | CDA_c.154+1015A>G | A/G |
|  | rs10916824 | CDA_c.154+3136T>C | T/T |
|  | rs4655226 | CDA_c.155-3137C>T | C/C |
|  | rs60369023 | CDA*3_c.208G>A(A70T) | G/G |
|  | rs1048977 | CDA_c.435C>T(T145=) | T/C |
| *DPYD* | |  |  |
|  | rs12132152 | DPYD_c.*21528C>T(3'UTR) | G/G |
|  | rs76387818 | DPYD_c.*5132C>T(3'UTR) | G/G |
|  | rs1801268 | DPYD*10_c.2983G>T(V995F) | C/C |
|  | rs67376798 | DPYD_c.2846A>T(D949V) | T/T |
|  | rs290852 | DPYD_c.2767-2165A>G | T/C |
|  | rs17471125 | DPYD_c.2767-5102A>G | T/T |
|  | rs1801267 | DPYD*9B_c.2657G>A(R886H) | C/C |
|  | rs147545709 | DPYD_c.2656C>T(R886C) | G/G |
|  | rs1760217 | DPYD_c.2623-38806T>C | A/G |
|  | rs7552825 | DPYD_c.2623-42576G>A | C/C |
|  | rs6656660 | DPYD_c.2622+9416C>A | T/G |
|  | rs2027056 | DPYD_c.2300-23051C>A | T/T |
|  | rs12140120 | DPYD_c.2300-23459G>A | C/C |
|  | rs1801160 | DPYD*6_c.2194G>A(V732I) | C/C |
|  | rs72728438 | DPYD_c.1974+75A>G | T/T |
|  | rs2152878 | DPYD_c.1906-5426A>G | T/T |
|  | rs12022243 | DPYD_c.1906-14763G>A | C/C |
|  | rs7548189 | DPYD_c.1906-19696G>T | C/C |
|  | rs4492658 | DPYD_c.1906-28506C>G | C/G |
|  | rs3918290 | DPYD*2_c.1905+1G>A(SpliceVariant) | C/C |
|  | rs3918289 | DPYD_c.1905C>G/T(N635K/N) | G/G |
|  | rs72549303 | DPYD*3_c.1898delC(P633Frameshift) | G/G |
|  | rs17376848 | DPYD_c.1896T>C(F632=) | A/A |
|  | rs3897854 | DPYD_c.1741-2527G>A | T/C |
|  | rs12136186 | DPYD_c.1741-16477A>G | T/C |
|  | rs17116806 | DPYD_c.1740+8030G>T | C/C |
|  | rs55886062 | DPYD*13_c.1679T>G(I560S) | A/A |
|  | rs1801159 | DPYD*5_c.1627A>G(I543V) | T/T |
|  | rs1801158 | DPYD*4_c.1601G>A(S534N) | C/C |
|  | rs2811219 | DPYD_c.1340-11501T>C | A/A |
|  | rs56038477 | DPYD_c.1236G>A(E412=) | C/C |
|  | rs78060119 | DPYD*12_c.1156G>T(E386X) | C/C |
|  | rs75017182 | DPYD_c.1129-5923C>G(SpliceVariant) | G/G |
|  | rs1042479 | DPYD_c.1074T>A(R358=) | A/A |
|  | rs1042478 | DPYD_c.1035T>C(F345=) | A/A |
|  | rs72549306 | DPYD*11_c.1003G>T(V335L) | C/C |
|  | rs2811196 | DPYD_c.851-18271A>G | T/T |
|  | rs10518636 | DPYD_c.851-38157C>T | A/G |
|  | rs2786507 | DPYD_c.850+26331C>T | A/G |
|  | rs45589337 | DPYD_c.775A>G(K259E) | T/T |
|  | rs6675198 | DPYD_c.756T>C(G252=) | A/A |
|  | rs1801266 | DPYD*8_c.703C>T(R235W) | G/G |
|  | rs12119882 | DPYD_c.680+2545T>C | A/A |
|  | rs115232898 | DPYD_c.557A>G(Y186C) | T/T |
|  | rs6670886 | DPYD_c.525G>A(S175=) | C/C |
|  | rs2297595 | DPYD_c.496A>G(M166V) | T/T |
|  | rs72549309 | DPYD*7_c.295_298delTCAT(F100Frameshift) | ATGA/ATGA |
|  | rs17378539 | DPYD_c.234-3075A>G | T/T |
|  | rs115632870 | DPYD_c.151-69G>A | C/C |
|  | rs1801265 | DPYD*9_c.85T>C(C29R) | A/A |
|  | rs80081766 | DPYD_c.62G>A(R21Q) | C/C |
|  | rs72549310 | DPYD_c.61C>T(R21X) | G/G |
|  | rs4970722 | DPYD_c.40-3123T>A | T/T |
| *ABCC3* | |  |  |
|  | rs9895420 | ABCC3_c.-260T>A(5'UTR) | A/A |
|  | rs1003355 | ABCC3_c.1583C>T(A528V) | C/C |
|  | rs4148416 | ABCC3_c.3039C>T(G1013=) | T/C |
|  | rs11568591 | ABCC3_c.3890G>A(R1297H) | G/G |
|  | rs2277624 | ABCC3_c.3942C>T(H1314=) | T/C |
|  | rs1051625 | ABCC3_c.4084C>G(L1362V) | C/C |
|  | rs1051640 | ABCC3_c.4509A>G(E1503=) | A/A |
| *ABCC4* | |  |  |
|  | rs4148555 | ABCC4_c.*1564A>T(3'UTR) | T/T |
|  | rs9516519 | ABCC4_c.*1372A>C(3'UTR) | T/G |
|  | rs1059754 | ABCC4_c.*1351T>C(3'UTR) | A/A |
|  | rs4148554 | ABCC4_c.*1282T>C(3'UTR) | A/A |
|  | rs1059751 | ABCC4_c.*879T>C(3'UTR) | A/A |
|  | rs4148553 | ABCC4_c.*694G>A(3'UTR) | C/C |
|  | rs4148551 | ABCC4_c.*311G>A(3'UTR) | T/C |
|  | rs3742106 | ABCC4_c.*38T>G(3'UTR) | A/A |
|  | rs9561765 | ABCC4_c.3870+3616C>T | G/G |
|  | rs11568695 | ABCC4_c.3609G>A(A1203=) | C/C |
|  | rs9556455 | ABCC4_c.3457-724C>T | A/G |
|  | rs9561778 | ABCC4_c.3366+1243C>A | G/G |
|  | rs1751034 | ABCC4_c.3348A>G(K1116=) | T/T |
|  | rs11568655 | ABCC4_c.3310T>C(L1104=) | A/A |
|  | rs1678387 | ABCC4_c.3211-2793A>G | C/C |
|  | rs1189466 | ABCC4_c.2844C>T(F948=) | G/G |
|  | rs1678339 | ABCC4_c.2712G>A(L904=) | C/C |
|  | rs16950650 | ABCC4_c.2456-7177G>A | C/C |
|  | rs3765534 | ABCC4_c.2269G>A(E757K) | C/C |
|  | rs72559753 | ABCC4_c.1892delT(L631Frameshift) | A/A |
|  | rs2274405 | ABCC4_c.969G>A(S323=) | C/C |
|  | rs2274406 | ABCC4_c.951A>G(R317=) | C/C |
|  | rs2274407 | ABCC4_c.912G>T(K304N) | A/C |
|  | rs899494 | ABCC4_c.669T>C(I223=) | G/G |
|  | rs11568658 | ABCC4_c.559G>T(G187W) | C/C |
|  | rs4148460 | ABCC4_c.511T>G(C171G) | A/A |
|  | rs7317112 | ABCC4_c.75-23516T>C | A/G |
|  | rs868853 | ABCC4_c.-1508G>A(5'UTR) | T/C |
| *ABCC5* | |  |  |
|  | rs1000002 | ABCC5_c.*3320C>T(3'UTR) | T/C |
|  | rs3805114 | ABCC5_c.*1366A>C(3'UTR) | T/T |
|  | rs562 | ABCC5_c.*1243G>A(3'UTR) | C/C |
|  | rs8180093 | ABCC5_c.4212+1040C>T | G/G |
|  | rs1053387 | ABCC5_c.4148C>A(T1383N) | G/G |
|  | rs3749442 | ABCC5_c.3624C>T(L1208=) | A/G |
|  | rs1053351 | ABCC5_c.3606C>A(Y1202X) | G/G |
|  | rs72551384 | ABCC5_c.3441_3442insC(V1147Frameshift) | -/- |
|  | rs939336 | ABCC5_c.1782C>T(C594=) | G/G |
|  | rs3805111 | ABCC5_c.1405-282C>T | G/G |
|  | rs3792581 | ABCC5_c.1296+421G>T | A/C |
|  | rs1053386 | ABCC5_c.1200C>T(S400=) | A/G |
|  | rs7636910 | ABCC5_c.1146A>G(Q382=) | T/T |
|  | rs2293001 | ABCC5_c.999+28G>A | T/C |
|  | rs4148572 | ABCC5_c.129+11553G>C | C/G |
|  | rs4148557 | ABCC5_c.-55-1549T>C | G/G |
| *ABCG2* | |  |  |
|  | rs2231164 | ABCG2_c.1738-46G>A | C/C |
|  | rs45605536 | ABCG2_c.1582G>A(A528T) | C/C |
|  | rs58818712 | ABCG2_c.1574T>G(L525R) | A/A |
|  | rs4148157 | ABCG2_c.1368-334C>T | G/G |
|  | rs2622628 | ABCG2_c.1195-834T>G | A/C |
|  | rs13120400 | ABCG2_c.1194+928A>G | T/T |
|  | rs3201997 | ABCG2_c.1000G>T(E334X) | C/C |
|  | rs41282401 | ABCG2_c.886G>C(D296H) | C/C |
|  | rs3116448 | ABCG2_c.742T>C(S248P) | A/A |
|  | rs1061018 | ABCG2_c.623T>C(F208S) | A/A |
|  | rs3116439 | ABCG2_c.564A>G(G188=) | T/T |
|  | rs2725256 | ABCG2_c.531+1215T>C | A/A |
|  | rs2231142 | ABCG2_c.421C>A(Q141K) | G/G |
|  | rs72552713 | ABCG2_c.376C>T(Q126X) | G/G |
|  | rs2231139 | ABCG2_c.369C>T(Y123=) | G/G |
|  | rs17731538 | ABCG2_c.204-1592C>T | G/G |
|  | rs17731799 | ABCG2_c.-19-7289C>A | T/G |
|  | rs2622604 | ABCG2_c.-20+614A>G | C/C |
|  | rs45630471 | ABCG2_c.-29A>G(5'UTR) | T/T |
|  | rs2231135 | ABCG2_c.-476T>C(5'UTR) | A/A |
|  | rs72554040 | ABCG2_rs72554040(5'UTR) | G/G |
| *SLC22A7* | |  |  |
|  | rs2651185 | SLC22A7_c.-1310T>C | T/T |
|  | rs36040909 | SLC22A7_c.979C>T(R327W) | C/C |
|  | rs35385682 | SLC22A7_c.1017C>A(R339=) | C/C |
|  | rs2651184 | SLC22A7_c.1221C>T(A407=) | C/C |
|  | rs2270860 | SLC22A7_c.1275C>T(S425=) | T/C |
|  | rs2841646 | SLC22A7_c.1277-67G>A | G/G |
|  | rs2242416 | SLC22A7_c.563A>G(I188T) | A/A |
| *SLC29A1* | |  |  |
|  | rs747199 | SLC29A1:c.-55+441G>C | G/G |
|  | rs9394992 | SLC29A1:c.29+913C>T | T/C |
|  | rs8187630 | SLC29A1_c.84G>A(P28=) | G/G |
|  | rs8187640 | SLC29A1_c.597G>A(S199=) | G/G |
|  | rs45573936 | SLC29A1_c.647T>C(I216T) | T/T |
|  | rs8187642 | SLC29A1_c.687G>A(L229=) | G/G |
|  | rs186556 | SLC29A1_c.864+4C>T | C/C |
|  | rs8187633 | SLC29A1_c.999T>C(C333=) | T/T |
|  | rs45458701 | SLC29A1_c.1171G>A(E391K) | G/G |
|  | rs760370 | SLC29A1:c.1260-201A>G | A/A |
|  | rs8187637 | SLC29A1_c.*46C>A | C/C |
|  | rs14623 | SLC29A1_c.*442G>A | G/G |
| *MTHFR* | |  |  |
|  | rs1476413 | MTHFR:c.1755+35G>A | C/C |
|  | rs4846051 | rs4846051 | A/A |
|  | rs1801131 | MTHFR:c:.1406A>C:p.Glu429Ala | A/A |
|  | rs1801133 | MTHFR:c.665C>T:p.Ala222Val | C/T |
|  | rs17421511 | MTHFR:c.710-1332C>T | G/G |
|  | rs17367504 | rs17367504 | A/A |
|  | rs1801131 + rs1801133 | 665C>T-1406A>C diplotype | CA-TA |
| *TYMS* | |  |  |
|  | rs59755869 | TYMS_c.298G>C(E100Q) | G/G |
|  | rs11540152 | TYMS_c.349T>C(F117L) | T/T |
|  | rs3786362 | TYMS_c.381A>G(E127=) | A/A |
|  | rs596909 | TYMS_c.470G>T(G157V) | G/G |
|  | rs11540153 | TYMS_c.500C>T(T167I) | C/C |
|  | rs151264360 | TYMS:c.*447_*452delTTAAAG | -/TTAAAG |
|  | rs2847153 | TYMS:c.280-499G>A | G/A |
|  | rs2853535 | TYMS:c.732+190C>G | G/G |

**Supplemental Figure 1**

**
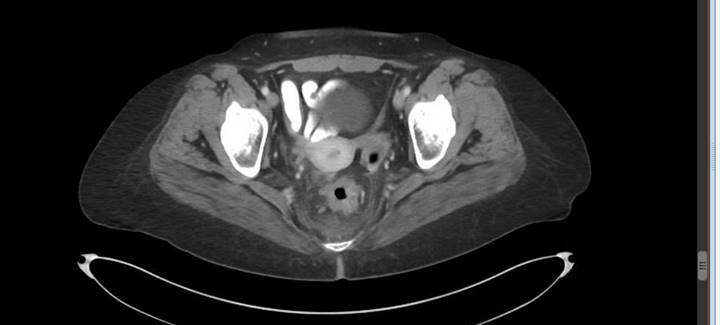
**

**Figure 1 Legend:** Axial image from preoperative CT scan demonstrating pelvic and peri-rectal inflammation.
